# Optimization of Fuel Cell Performance Using Computational Fluid Dynamics

**DOI:** 10.3390/membranes11020146

**Published:** 2021-02-20

**Authors:** Tabbi Wilberforce, Oluwatosin Ijaodola, Ogungbemi Emmanuel, James Thompson, Abdul Ghani Olabi, Mohammad Ali Abdelkareem, Enas Taha Sayed, Khaled Elsaid, Hussein M. Maghrabie

**Affiliations:** 1 Mechanical Engineering and Design, School of Engineering and Applied Science, Aston University, Aston Triangle, Birmingham B4 7ET, UK; aolabi@sharjah.ac.ae; 2 Institute of Engineering and Energy Technologies, University of the West of Scotland, Glasgow G72 0LH, UK; oluwatosin.ijaodola@uws.ac.uk (O.I.); emmanuel.ogungbemi@uws.ac.uk (O.E.); james.thompson@uws.ac.uk (J.T.); 3 Department of Sustainable and Renewable Energy Engineering, University of Sharjah, P.O. Box 27272, Sharjah, United Arab Emirates; mabdulkareem@sharjah.ac.ae; 4 Center for Advanced Materials Research, University of Sharjah, P.O. Box 27272, Sharjah, United Arab Emirates; e.kasem@mu.edu.eg; 5 Chemical Engineering Department, Minia University, Minya 61111, Egypt; 6 Chemical Engineering Department, Texas A&M University, College Station, TX 77843-3122, USA; khaled.elsaid@qatar.tamu.edu; 7 Department of Mechanical Engineering, Faculty of Engineering, South Valley University, Qena 83521, Egypt; Hussein_mag@eng.svu.edu.eg

**Keywords:** PEM fuel cell, serpentine bipolar plate, polarization curve, stainless steel, copper

## Abstract

A low cost bipolar plate materials with a high fuel cell performance is important for the establishment of Proton Exchange Membrane (PEM ) fuel cells into the competitive world market. In this research, the effect of different bipolar plates material such as Aluminum (Al), Copper (Cu), and Stainless Steel (SS) of a single stack of proton exchange membrane (PEM) fuel cells was investigated both numerically and experimentally. Firstly, a three dimensional (3D) PEM fuel cell model was developed, and simulations were conducted using commercial computational fluid dynamics (CFD) ANSYS FLUENT to examine the effect of each bipolar plate materials on cell performance. Along with cell performance, significant parameters distributions like temperature, pressure, a mass fraction of hydrogen, oxygen, and water is presented. Then, an experimental study of a single cell of Al, Cu, and SS bipolar plate material was used in the verification of the numerical investigation. Finally, polarization curves of numerical and experimental results was compared for validation, and the result shows that Al serpentine bipolar plate material performed better than Cu and SS materials. The outcome of the investigation was in tandem to the fact that due to adsorption on metal surfaces, hydrogen molecules is more stable on Al surface than Cu and SS surfaces.

## 1. Introduction

**Figure 1:**
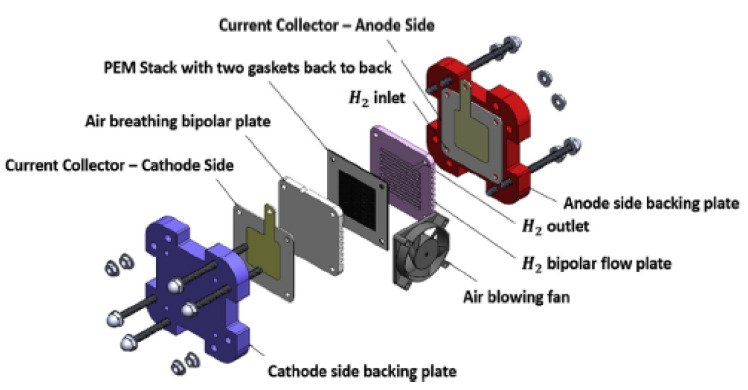
Exploded view of a single PEM fuel cell [6].

Proton exchange membrane (PEM) fuel cells have grown as a possible energy source, over the last decade. Researchers throughout the world are concentrating on how to optimize the system to make it cost-competitive with other sources of energies currently available in the market [1,2,3]. PEM fuel cells are environmental friendly with a high-efficiency power source and are not bound by carnot efficiency. They are viewed as one of the promising candidates to be used for electric vehicles due to their qualities of zero pollution, high power density, quick start-up, and low operating temperature [4,5]. A PEM fuel cell is mainly made up of an anode, cathode, gas diffusion layer, catalyst layer, and electrolyte membrane, as shown in Figure 1. They can be used for distribution of power systems, aerospace, and submarines applications. A practical voltage of a single operating cell is about 0.8V. The desired cell voltages can be generated by the connection of cells in series; this can be achieved by putting a high conductive material (such as a bipolar plate) between a two parallel membrane electrode assembly (MEA). A bipolar plate is a vital component of a fuel cell, and it accounts for about 40–45% of the total cost, and more than 70% of the total weight [6,7,8]. As a consequence of this, notable research and development work is being conducted to reduce the size, lower the cost, and improve the lifetime and performances of fuel cells. The bipolar plates function as a current conductor, facilitate water and heat management, provide structural support for the entire stack, and provide pathways for reactant gases within the cell [9].

**Figure 2:**
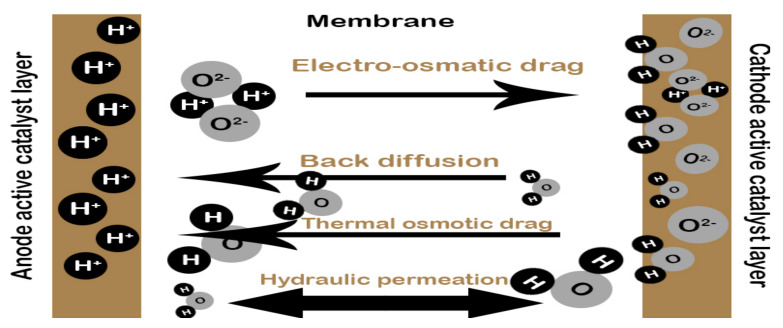
Water transport mechanism in a PEM fuel cell.

Meanwhile, the bipolar plate helps in water transportation within the cell, which plays a role in the contribution of water management, which is influenced by the mechanisms of water transportation of a fuel cell, as shown in Figure 2. Hence, flow channels on the bipolar plate do play an essential aspect in the cell, to keep an excellent overall performance.

Tabbi et al. [10] propose modifications to some conventional design of bipolar late flow design of PEM fuel cells. The investigation was conducted by using computational fluid dynamics (CFD) Ansys CFX to study various designs and their effect on pressure drops by varying the flow rate for each design modeled. They concluded that modifications of existing conventional designs, using a method that is similar to a diesel injection system, did reduce the pressure drop, which will contribute to the overall improvement of PEM fuel cell performance. 

Hong et al. [11] studied the challenges facing uniform flow distribution in fuel cell applications. They present a novel method for uniform flow field channel distribution, for which multiple levels of flow channel bifurcations were examined, to distribute flow uniformly from n into 2n levels flow field channels of bifurcation. CFD fluent software was used to study flow channel bifurcation dimensions and structure effects on the uniformity of flow distribution in the fuel cell. They found out that the length of flow channel against the width, in the straight part after a 90-degree turn, does have a crucial effect on the uniformity of flow distribution. Additionally, the curvature of flow channels at a 90 degree turning area and round corners are essential for flow field channel uniformity as a result of the low-pressure loss. Lung and leaf design flow field channels were proposed by Jason et al. [12]. This bio-inspired design was evaluated using numerical simulation (COSMOL Multiphysics 3.4) to study the effect on fuel cell performance. The maximum operating conditions for both designs was a cell temperature between 65 °C and 75 °C, with the backpressure of 2 atm and 100% relative humidity. Under these cell operating conditions, it was seen that there was a notable improvement in the pressure drop, peak power density, and overall performance than the conventional flow channels. Mohammad and Behzad [13] examine the effects of flow field channels dimensions on fuel cell performance. The simulation result demonstrated that if the channel width is increased, then the limiting current density will improve. A CFD investigation on a PEM fuel cell was conducted in order to examine the performance of three different conventional flow channels modify to tubular plates by Sierra et al. [14]. The fluid dynamics equation solution, CFD electrochemical model and different variables of distribution contours were obtained. The distribution contours permit local analysis of transport occurrence, which shows that the tubular design display more uniformity in distributions of oxygen concentration, hydrogen, current density, and pressure than a conventional flow channels design. It was concluded that the tubular design that has straight channels shows the least pressure drop among the channels. Sukkee and Wang [15] presented an investigation of a three dimensional (3D) interplay between electrochemical kinetics and mass transport of interdigitated and straight flow field channels using a computational fuel cell dynamics (CFCD) model. The model results point out that forced convection effects, caused by the interdigitated flow channel, significantly improves the mass transport of oxygen and removes water from the catalyst layer, compared to a straight flow channel. 

Yousef et al. [16] investigated how effective a novelty compound flow field channel design affects the performance of a PEM fuel cell by employing a CFD approach. They reach a conclusion from the simulation result that this compound design can perform better than the conventional design due to better discharge of water.

In this paper, a serpentine bipolar plate made up of aluminum, Copper, and Steel materials with an active area of 25 cm^2^ active area of a PEM fuel cell flow field channels was developed using SOLID WORKS 2016 version software and imported into ANSYS FLUENT 18.0 licensed software for simulation. 

The parameters such as temperature differences, the variation of pressure in flow field channels, and mass fraction for reactants and water was predicted numerically, alongside with fuel cell performance using polarisation curves. In order to affirm the numerical results, experimental works were conducted and the simulation results were validated with the experimental results. The validation of the numerical data will guide to design and develop a practical PEM fuel cell.

## 2. Geometry Design

**Figure 3:**
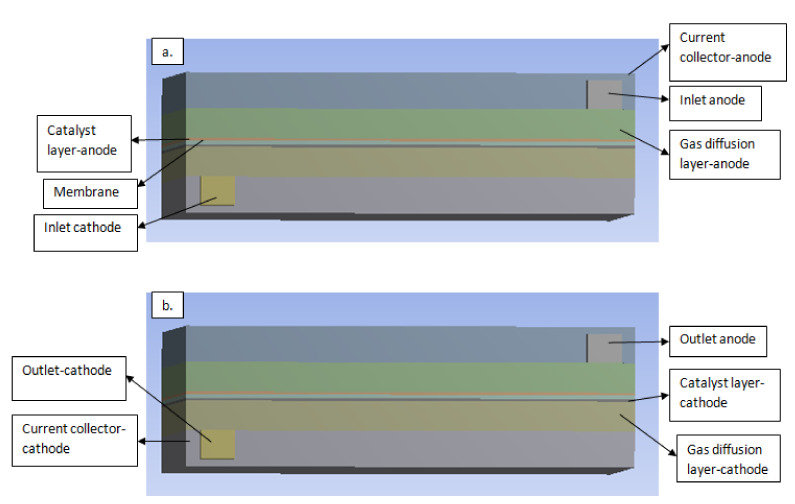
A three-dimensional view of the single PEM fuel cell and its components. (**a**) Inlet channel (**b**) outlet channel.

The geometry model was built using SOLID WORKS 2016 version software. Then the geometry was generated in this software and relevant zones which are used for the modeling domains such as current collectors, flow field channels, gas diffusion layers, catalyst layers and membrane for both, as shown in Figure 3. After, the grid was exported to ANSYS FLUENT 18.0 licensed software, where the meshing and complete set of the module was solved. ANSYS provides an additional add-on PEM fuel cell module, which was used in solving the fluid-based equation for the fuel cell. The models were read into the FLUENT application, then various specifications and boundary conditions were set for each zone. 

This numerical model domain is a single cell geometry. The reactant gases for this model is humidified hydrogen and air. The mass flow inlet was controlled using stoichiometry numbers of 0.6 at the anode side and 0.4 at the cathode side. The operating temperature was 323, 333, and 353 K. While the operating pressure was 150, 200, 250 kPa, respectively. The active area for the model is 5 × 5 cm^2^; the channels are 2 mm in width and 2 mm in depth. The rib width is 2 mm. Table 1 and Table 2 shows the geometry properties and parameters for the simulation.

### 2.1. Computational Domain

A three dimensional model for a PEM fuel cell was used in this study. The geometry model of the single fuel cell with parts consisting of anode and cathode current collectors, gas flow channels, Gas diffusion layers (GDLs), Catalyst layers (CLs), and the membrane was merged and meshed through the FLUENT mesh interfaces operation. It was carried out using the automatic meshing method, and the size function of the model was set to uniformity.

The relevance centre were set as fine and the element order was linear. The transition was set to fast. These setting end up to have 59,160 nodes and 85,173 elements. The total computational domain consists of 41,999 grid cells.

### 2.2. Boundary Conditions

The mesh was exported to FLUENT setup function to start iterations after it was found suitable. The boundary conditions are defined as follows: constant anode and cathode mass flow rate at both the inlet gas channels and constant pressure state of the channel’s outlet. The model external surfaces are assumed as a wall. The Multi-grid cycle function was altered to F-cycle for simulation stabilization in the process of ANSYS FLUENT PEM fuel cell simulation, and the method for all specified equations. The F-cycle was selected in order to intensify the parallel computing. Water saturation, species concentration, protonic and electronic potential used bi-conjugate gradient stabilization (BCGSTAB), this is because it provides better convergence results when compared to any other stabilization processes. The anode and cathode region of the reactant inlet conditions are mass flow inlet category; the pressure outlet was selected as the gases outlet. The operational temperature and pressure for each bipolar plate materials were compared as shown in Table 2, for the anode and cathode region of the fuel cell. The numerical proceedings for every parameter in the computational domain for the boundary conditions and cell voltage at the cathode are set with the transport and momentum equations for all the species are resolved. In the computational domain, all species distribution are acquired, then the process is repeated until convergence is obtained.

## 3. Mathematical Modelling

The governing set of equations used in solving the computational model in this study was discussed by Sierra et al. [14]. These equations set listed in Table 3 commensurate to energy, species, charge, mass conservation, momentum charge transport equations. According to Bladimir et al. [17]. The mass flux diffusion J*_i_*_,__ϵ_ of i species along ϵ direction is given by:

(1) $$J_{i,\;\epsilon }=-\rho D_{i}\;\frac{\partial Y_{i}}{\partial \in }$$

Um et al. [18] reported the diffusion coefficient D*_i_* as:

(2) $$D_{i}\;\varepsilon^{1.5}D_{i}^{O}\left(\frac{P_{o}}{P}\right)\left(\frac{T}{T_{o}}\right)3/2$$

where $D_{i}^{O}$ is diffusivity of *i* species at reference pressure and temperature, of 1atm and 300K, respectively. The membrane electrolyte conductivity depends on it water content and temperature [18]. Water generated from the electrochemical reaction diffuses towards the anode region and water transport in the membrane is mainly due to back diffusion and electro-osmotic force. Springer et al. [19] proposed the ionic conductivity of a PEM fuel cell with the expression described below:

(3) $$\sigma_{\mathrm{mem}}\;={\;}_{\epsilon }(0.514\lambda -0.326)\exp[1268(\frac{1}{303}-\frac{1}{T})]$$

The membrane porosity is ε, and λ is the amount of water content in the membrane, which is defined as:

(4) $$\lambda \;=\;\left\{\begin{array}{cc} 0.043+17.18a_{k}-39.85a+36a_{k}^{3} & a_{k}<1\\ 14+1.4\left(a_{k}-1\right) & a_{k}<1 \end{array}\right.$$

The liquid water activity is $a_{k}$ and it is calculated with the expressions:

(5) $$a_{k}=\frac{P_{vap}}{P_{sat}}$$

Springer et al. modeled the saturation pressure as:

(6) $$\log_{10}\;P{\mathrm{sat}}_{\;}{=}_{\;}-2.1794\;+\;0.02953\theta \;-\;9.1837\;\times \;10^{-5}\theta^{2}\;+\;1.4454\;\times \;10^{-7}\theta^{3}$$

where θ = T − 273.16.

The two potential equation solved in the model is electron transport through solid materials and ionic transportation. These potential equations are expressed as:

∇·(σ_sol_ ∇ø_sol_) + R_sol_ = 0 (7)

∇·(σ_mem_ ∇ø_mem_) + R_mem_= 0 (8)

The source terms or transfer current within the catalyst layer are nonzero only and are resolved as R_sol_ = -R_a_ at the anode side of the solid phase and R_sol_ = -R_c_ at the cathode side. As for the membrane phase, R_mem_ = +R_a_ at the anode side and R_mem_ = -R_c_ at the cathode side.

R_a_ and R_c_ are the current exchange densities and they are calculated using the Butler–Volmer equation:

(9) $$R_{a}=i_{a}^{ref}\left(\frac{H_{2}}{H_{2\;ref}}\right)\;{}^{\gamma a}\;\left[\exp\left(\frac{\alpha_{a}F{ƞ}_{a}}{RT}\right)-\exp\left(-\frac{\alpha_{a}F{ƞ}_{a}}{RT}\right)\right]$$

(10) $$R_{c}=i_{c}^{ref}\left(\frac{O_{2}}{O_{2\;ref}}\right)\;{}^{\gamma c}\;\left[\exp\left(\frac{\alpha_{c}F{ƞ}_{c}}{RT}\right)-\exp\left(-\frac{\alpha_{c}F{ƞ}_{c}}{RT}\right)\right]$$

where i is the concentration at the electrode-electrolyte interface, i_ref_ is reference concentration, γ is concentration coefficient, α is transfer coefficient, ɳ is activation losses, F is Faraday constant. The anode and cathode over-potentials are related to the solid phase potential fields, and the membrane, ø_sol_ and ø_mem_ are given as:

ɳ_a_ = ø_sol_ − ø_mem_ (11)

ɳ_c_ = ø_sol_ − ø_mem_ − V_oc_ (12)

where V_oc_ is open-circuit voltage, as stated by Um et al. [20]

V_oc_ = 0.0025T + 0.2329 (13)

In a fuel cell, the open-circuit voltage symbolizes the reversible operating condition of the cell. This variable is a function of concentration and temperature due to the low operating temperatures of the PEM fuel cell. However, a fuel cell irreversible operating voltage is defined as:

V_oc_ = V_oc_ − ɳ_act_ − ɳ_ohm_ − ɳ_conc_ (14)

where ɳ_act_ is activation over-potential, which represents the irreversibility in the cell as a results of the energy losses during the chemical activation reactions; ɳ_ohm_ is ohmic over-potential, this is the irreversibility caused from the flow of ions across the electrolyte, and electrons across the current collectors and electrodes; while the ɳ_conc_ is mass transport or concentration over-potential, it occurs due to the high current density operation of the fuel cell [21]. When the energy demand conditions are high, then the electrochemical reactions consumption becomes faster.

**Table 3:** A summary of the fuel cell model governing equation.

Governing Equations	Mathematical Expressions	Ref.
Continuity	$\frac{\partial \left(\rho u\right)}{\partial x}$ + $\frac{\partial \left(\rho v\right)}{\partial y}$ + $\frac{\partial \left(\rho w\right)}{\partial z}$ = $S_{m}$	[22]
Momentum transport	$u\frac{\partial \left(\rho u\right)}{\partial x}$ + $v\frac{\partial \left(\rho v\right)}{\partial y}$ + $w\frac{\partial \left(\rho w\right)}{\partial z}$ = $-\;\frac{\partial P}{\partial x}+\frac{\partial }{\partial x}\left(\mu \frac{\partial u}{\partial x}\right)+$ $\frac{\partial }{\partial y}\left(\mu \frac{\partial u}{\partial y}\right)+\frac{\partial }{\partial z}\left(\mu \frac{\partial u}{\partial z}\right)+S_{px}$ $u\frac{\partial \left(\rho v\right)}{\partial x}$ + $v\frac{\partial \left(\rho v\right)}{\partial y}$ + $w\frac{\partial \left(\rho v\right)}{\partial z}$ = $-\;\frac{\partial P}{\partial y}+\frac{\partial }{\partial x}\left(\mu \frac{\partial v}{\partial x}\right)+$ $\frac{\partial }{\partial y}\left(\mu \frac{\partial v}{\partial y}\right)+\frac{\partial }{\partial z}\left(\mu \frac{\partial v}{\partial z}\right)+S_{py}$ $u\frac{\partial \left(\rho w\right)}{\partial x}$ + $v\frac{\partial \left(\rho w\right)}{\partial y}$ + $w\frac{\partial \left(\rho w\right)}{\partial z}$ = $-\;\frac{\partial P}{\partial z}+\frac{\partial }{\partial x}\left(\mu \frac{\partial w}{\partial x}\right)+$ $\frac{\partial }{\partial y}\left(\mu \frac{\partial w}{\partial y}\right)+\frac{\partial }{\partial z}\left(\mu \frac{\partial w}{\partial z}\right)+S_{pz}$	[23]
Energy	$u\frac{\partial \left(\rho CT\right)}{\partial x}$ + $v\frac{\partial \left(\rho CT\right)}{\partial y}$ + $w\frac{\partial \left(\rho CT\right)}{\partial z}$ = $\frac{\partial }{\partial x}\left(k\frac{\partial T}{\partial x}\right)+$ $\frac{\partial }{\partial y}\left(k\frac{\partial T}{\partial y}\right)+\frac{\partial }{\partial z}\left(k\frac{\partial T}{\partial z}\right)+\;S_{h}$	[24]
Hydrogen transport (anode region)	$u\frac{\partial \left(\rho Y_{H_{2}}\right)}{\partial x}$ + $v\frac{\partial \left(\rho Y_{H_{2}}\right)}{\partial y}$ + $w\;\frac{\partial \left(\rho Y_{H_{2}}\right)}{\partial z}=$ $\frac{\partial \left(J_{x,H_{2}}\right)}{\partial x}$ + $\frac{\partial \left(J_{y,H_{2}}\right)}{\partial y}$ + $\frac{\partial \left(J_{z,H_{2}}\right)}{\partial z}$ $+S_{H_{2}}$	[25]
Water transport (anode region)	$u\frac{\partial \left(\rho Y_{\;aw}\right)}{\partial x}$ + $v\frac{\partial \left(\rho Y_{\;aw}\right)}{\partial y}$ + $w\;\frac{\partial \left(\rho Y_{aw}\right)}{\partial z}=$ $\frac{\partial \left(J_{x,aw}\right)}{\partial x}$ + $\frac{\partial \left(J_{y,aw}\right)}{\partial y}$ + $\frac{\partial \left(J_{z,aw}\right)}{\partial z}$$\;+S_{aw}$	[26]
Oxygen transport (cathode region)	$u\frac{\partial \left(\rho Y_{O_{2}}\right)}{\partial x}\;$+$\;v\frac{\partial \left(\rho Y_{O_{2}}\right)}{\partial y}\;$+$w\;\frac{\partial \left(\rho Y_{O_{2}}\right)}{\partial z}=$ $\frac{\partial \left(J_{x,O_{2}}\right)}{\partial x}\;$+$\;\frac{\partial \left(J_{y,\;\;O_{2}}\right)}{\partial y}\;$+$\;\frac{\partial \left(J_{z,\;\;O_{2}}\right)}{\partial z}$$\;+S_{O_{2}}$	[27]
Water transport (cathode region)	$u\frac{\partial \left(\rho Y_{cw}\right)}{\partial x}\;$+$\;v\frac{\partial \left(\rho Y_{cw}\right)}{\partial y}\;$+$w\;\frac{\partial \left(\rho Y_{cw}\right)}{\partial z}=$ $\frac{\partial \left(J_{x,\;\;cw}\right)}{\partial x}\;$+$\;\frac{\partial \left(J_{y,\;\;cw}\right)}{\partial y}\;$+$\;\frac{\partial \left(J_{z,\;\;cw}\right)}{\partial z}$$\;+S_{cw}$	[28]
Source terms	S_m_ = $S_{H_{2\;}}+\;S_{aw}$ S_m_ = $S_{O_{2\;}}+\;S_{cw}$	[29]
	S_px_ = −$\frac{\mu \;u}{k}$ S_py_ = −$\frac{\mu \;v}{k}$ S_pz_ = −$\frac{\mu \;w}{k}$	[30]
	$\vec{J_{i}}$ = −$\rho \nabla \cdot y_{i}$	[31]
	S_h_ = I^2^R_ohm_ + h_react_ + ${ƞ}_{act}\;R_{an,ca}$	[32]
	$S_{H_{2}}=-\frac{M_{H_{2}}}{2F}R_{\mathrm{an}}$	[33]
	$S_{\mathrm{aw}}$ $=-\frac{M_{H_{2}}O}{F}R_{\mathrm{an}}$	[34]
	$S_{O_{2}}=-\frac{M_{O_{2}}}{4F}R_{\mathrm{ca}}$	[35]
	$S_{\mathrm{cw}}=-\frac{M_{H_{2}O}}{2F}R_{\mathrm{ca}}$	[36]
Charge transport	∇·(σ_sol_ ∇ø_sol_) + R_sol_ = 0 ∇·(σ_mem_ ∇ø_mem_) + R_mem_= 0	[37] [38]

## 4. Results and Discussion

### 4.1. Effects of Operating Temperature Variation

The operating temperature does play a vital role in improving fuel cell performance. In PEM fuel cell, increasing the operating temperature is beneficial in enhancing electrochemical reaction, ionic transport rate, and the removal of water from the fuel cell. Similarly, the fuel cell temperature can affect membrane life span and performance because if the membrane gets dry and stay like that for a long time, this may result in rupturing of the membrane. When observing the impact of the operating temperature on the cell performance, other parameters were kept constant while only the temperature was varied. 

**Figure 4:**
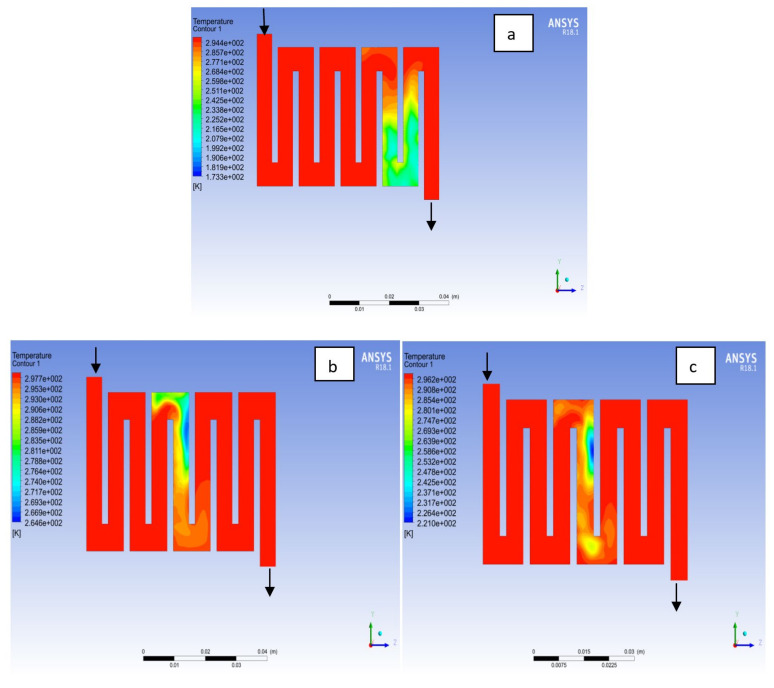
Temperature distribution at the anode region (GDL/CL) for each material at 298K: (**a**) Aluminum, (**b**) Copper, (**c**) Steel.

**Figure 5:**
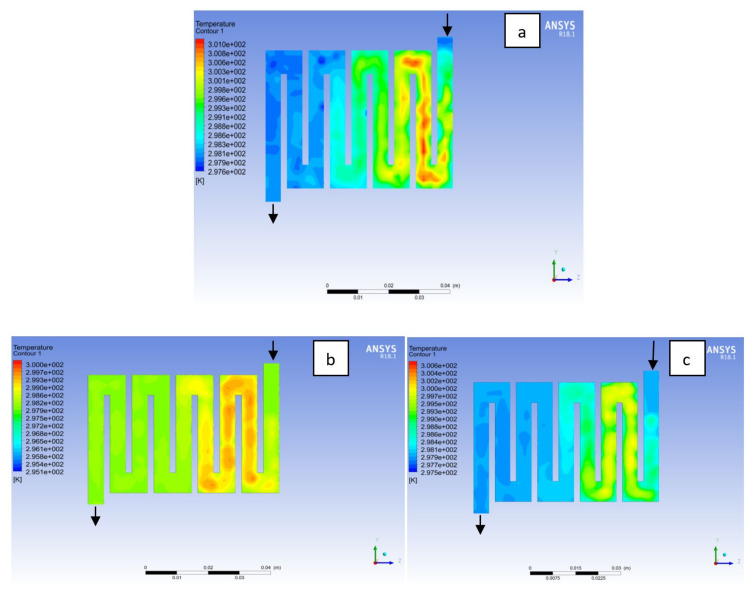
Temperature distribution at the cathode region (GDL/CL) for each material at 298K: (**a**) Aluminum, (**b**) Copper, (**c**) Steel.

**Figure 6:**
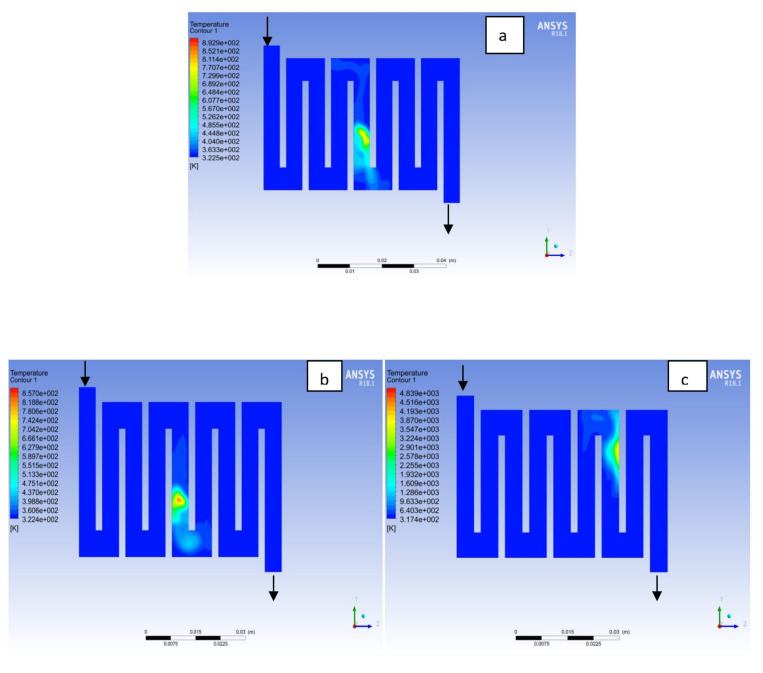
Temperature distribution at the anode region (GDL/CL) for each material at 323K: (**a**) Aluminum, (**b**) Copper, (**c**) Steel.

**Figure 7:**
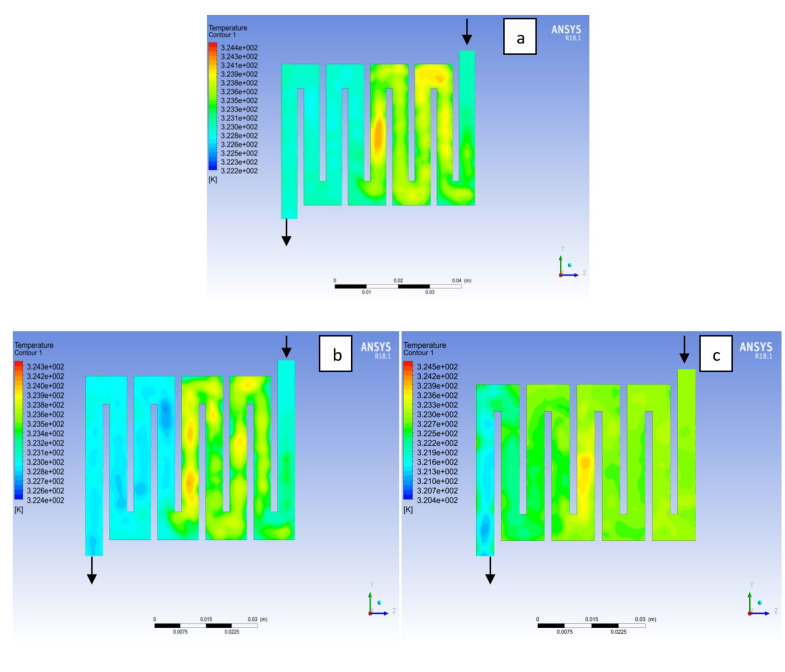
Temperature distribution at the cathode region (GDL/CL) for each material at 323K: (**a**) Aluminum, (**b**) Copper, (**c**) Steel.

**Figure 8:**
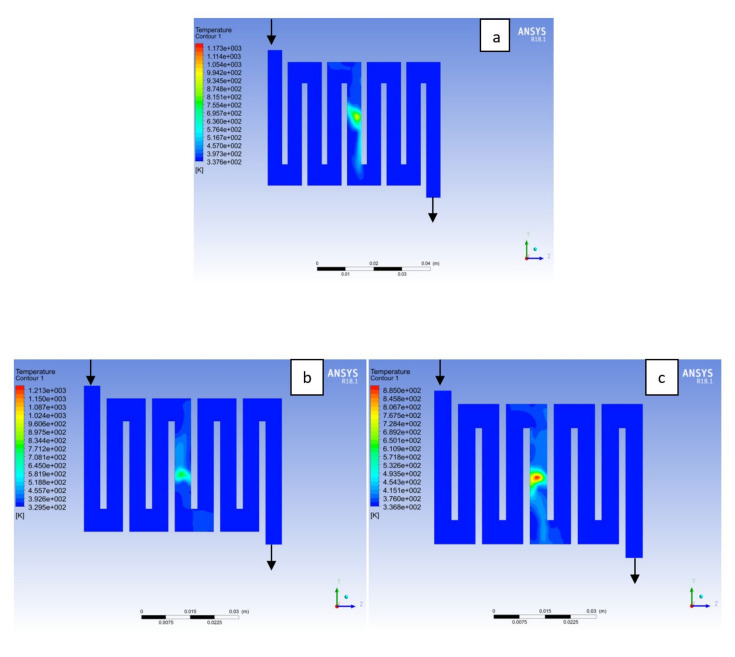
Temperature distribution at the cathode region (GDL/CL) for each materials at 338K: (**a**) Aluminum, (**b**) Copper, (**c**) Steel.

**Figure 9:**
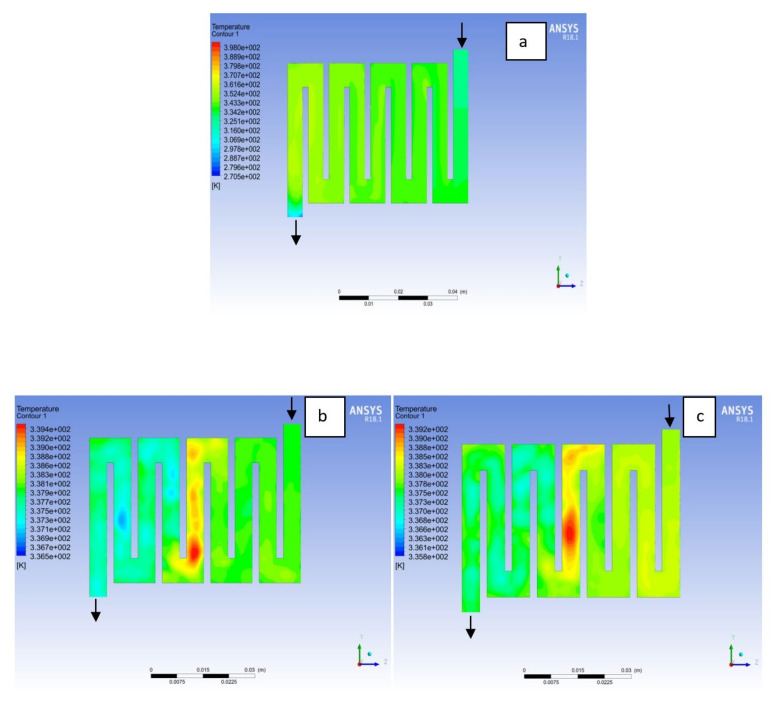
Temperature distribution at the cathode region (GDL/CL) for each material at 338K: (**a**) Aluminum, (**b**) Copper, (**c**) Steel.

The contours for temperature distribution in the channels for aluminium, copper and steel materials are shown in Figure 4, Figure 5, Figure 6, Figure 7, Figure 8 and Figure 9, respectively, for both anode and cathode side at temperatures of 298, 323, and 338 K at 0.7 V. The temperature distribution increases with increase in temperature. It can be observed at the interface between the catalyst layer and membrane in the anode region of each material that the temperature distribution is slightly different across each flow field channels and having temperature variations between 1 and 6 K. However, it was noticed that the highest temperature is at the cathode side for various materials temperature distribution in the channel due to electrochemical reaction and ohmic heating taking place. It is observed for Al material that the temperature at 338 K rises to 994 K, which is the highest temperature distribution variation, while at temperature 298 K steel materials has the lowest temperature distribution variation of 221 K among all other materials. The 3D numerical simulation temperature contours result shows that reactant adsorption on material surfaces has a significant effect on cell performance.

### 4.2. Effects of Operating Pressure Variation

**Figure 10:**
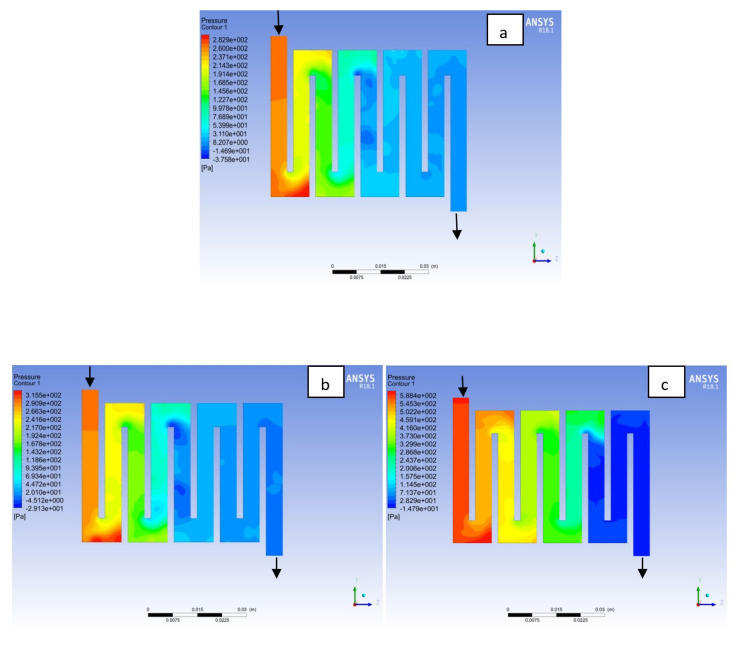
Pressure distribution at the anode region (GDL/CL) for each material with temperature 323 K at 1.5 bar: (**a**) Aluminum, (**b**) Copper, (**c**) Steel.

**Figure 11:**
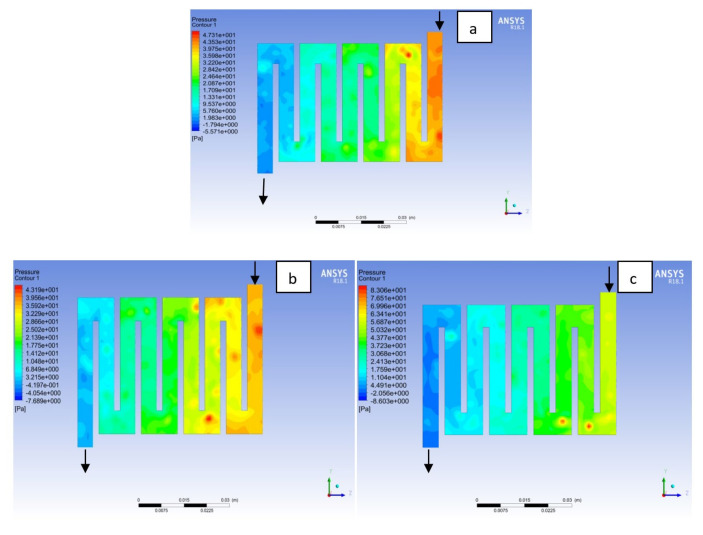
Pressure distribution at the cathode region (GDL/CL) for each material with temperature 323 K at 1.5 bar: (**a**) Aluminum, (**b**) Copper, (**c**) Steel.

**Figure 12:**
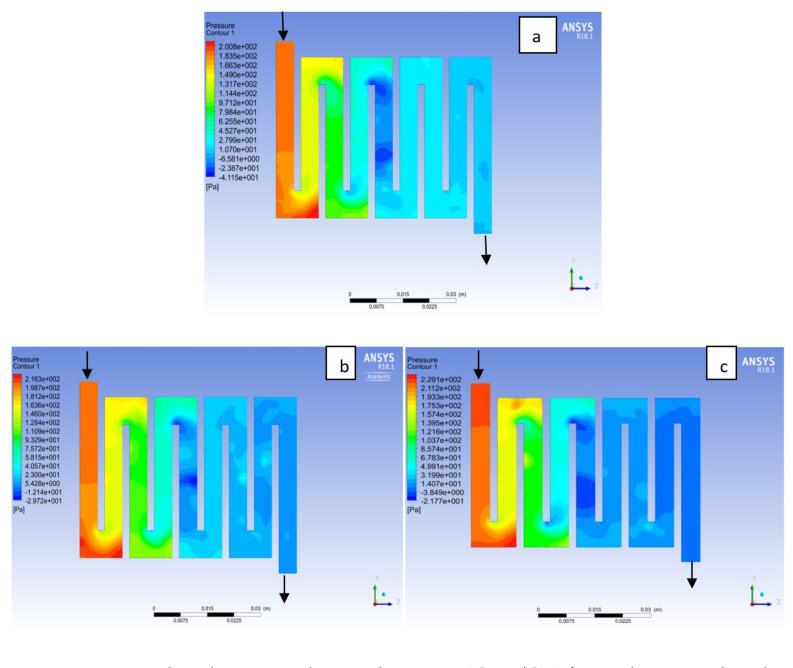
Pressure distribution at the anode region (GDL/CL) for each material with temperature 323 K at 2.0 bar: (**a**) Aluminum, (**b**) Copper, (**c**) Steel.

**Figure 13:**
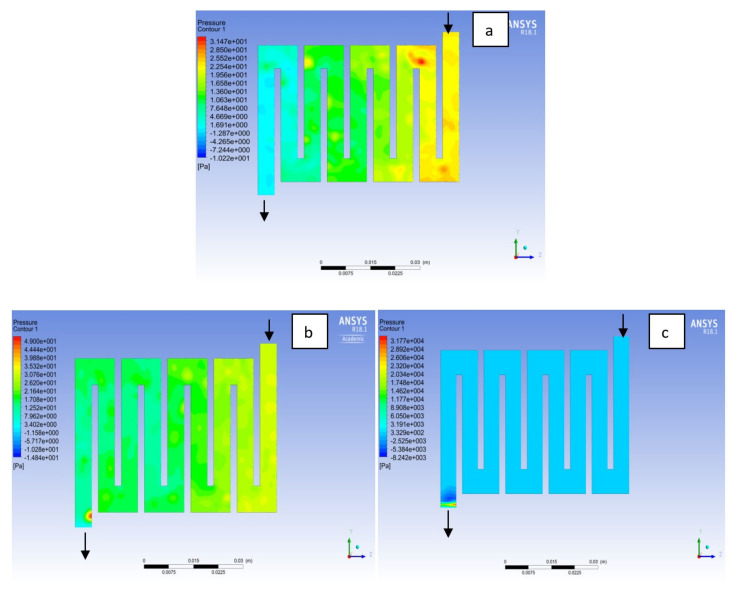
Pressure distribution at the cathode region (GDL/CL) for each material with temperature 323 K at 2.0 bar: (**a**) Aluminum, (**b**) Copper, (**c**) Steel.

**Figure 14:**
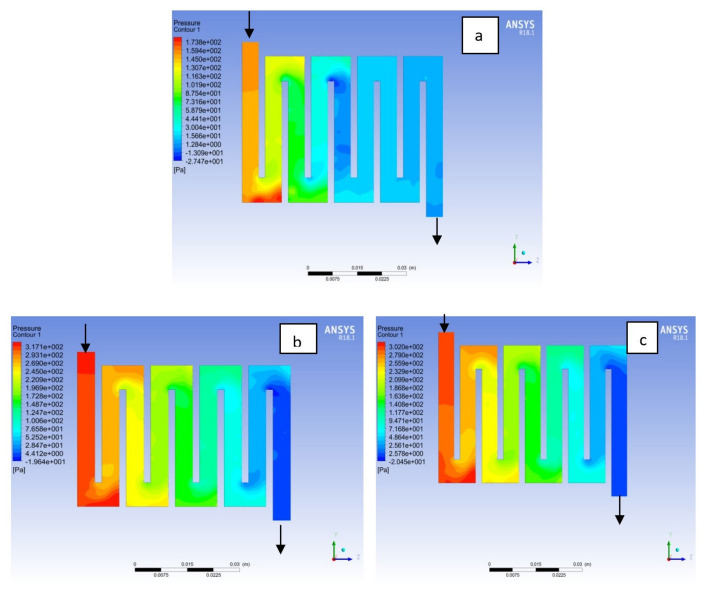
Pressure distribution at the anode region (GDL/CL) for each material with temperature 323 K at 2.5 bar: (**a**) Aluminum, (**b**) Copper, (**c**) Steel.

**Figure 15:**
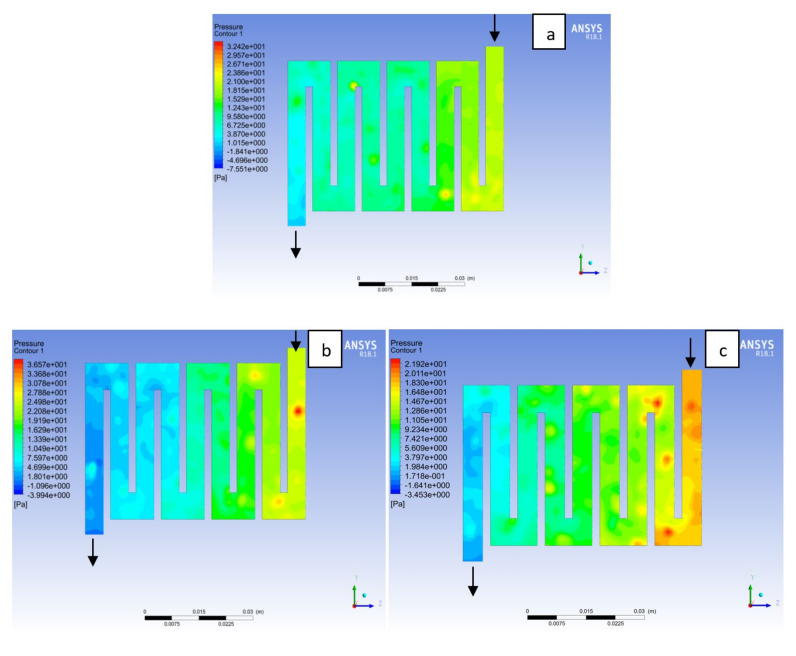
Pressure distribution at the cathode region (GDL/CL) for each material with temperature 323 K at 2.5 bar: (**a**) Aluminum, (**b**) Copper, (**c**) Steel.

Fuel cell operation could be at ambient pressure or pressurised state. Increase in pressure improves cell performance as stated by Yuh and Ay [27]. The reactant inflow pressure is always higher than the outflow pressure as a result of pressure drop within the flow field channels. Among the significant parameter that needs to be taken into account when designing a PEM fuel cell is pressure drop. The flow field channel is often the place where the pressure drop does take place and it does affect the electrochemical reaction of the fuel cell. Figure 10, Figure 11, Figure 12, Figure 13, Figure 14 and Figure 15 show the pressure drop at both anode and cathode channel at 1.5, 2.0, and 2.5 bar, respectively. The pressure drop is more at the inlet flow and gradually decreases along the outlet flow channel for all the bipolar plate materials. A similar observation was also stated by Alizadeh et al. [29]. It is observed that Al material has the least significant pressure drop at a different pressure range of the fuel cell than Cu and SS bipolar plate material. Besides bending and frictional losses, the adsorption of the reactants at the surface of the bipolar plate materials have contributed to the results. Moreover, a comparison between the anode and the cathode for each design revealed that the pressure at the anode decreases as the voltage decreases because the hydrogen must satisfy the current demanded by the cell. On the contrary, the gas pressure at the cathode increases as the voltage decreases due to the water generation in the electrode that causes a pressure rising in the flow channels.

### 4.3. Mass Fraction

**Figure 16:**
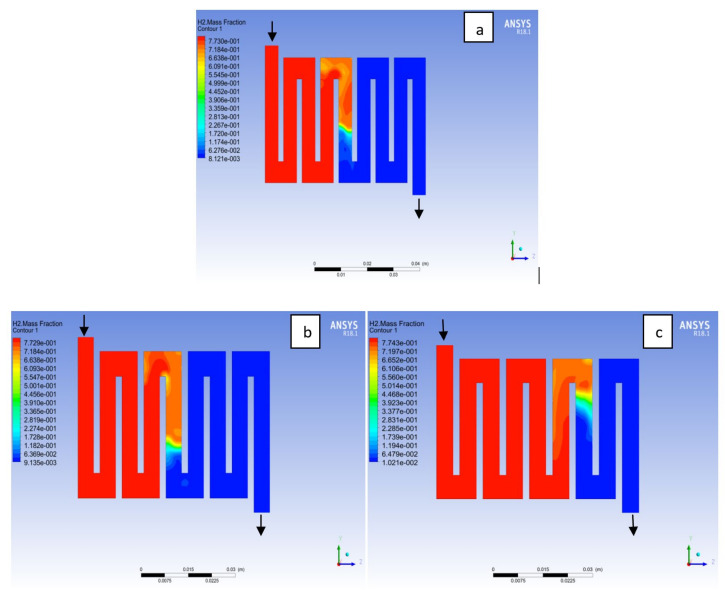
Contours of hydrogen mass fraction at the anode region (GDL/CL) for each material with temperature 323 K at pressure 1.5 bar: (**a**) Aluminum, (**b**) Copper, (**c**) Steel.

**Figure 17:**
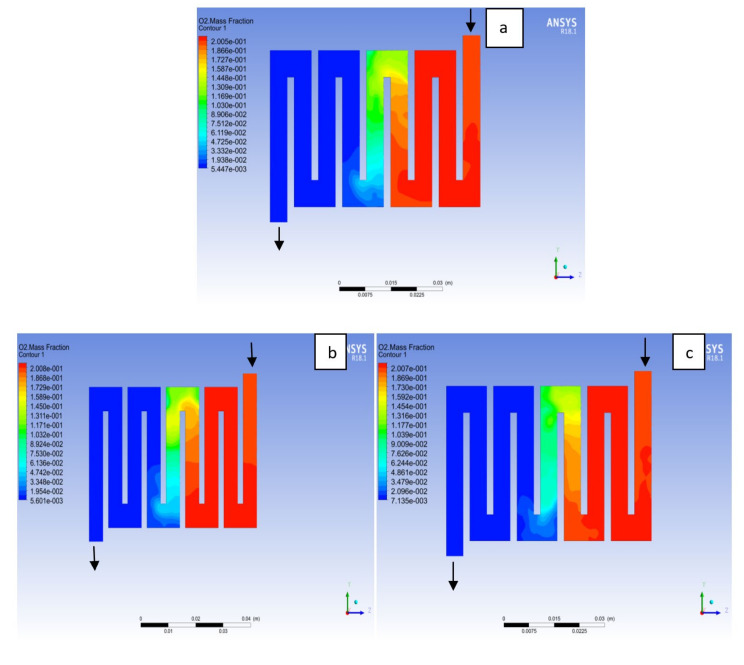
Contours of oxygen mass fraction at the cathode region (GDL/CL) for each material with temperature 323 K at pressure 1.5 bar: (**a**) Aluminum, (**b**) Copper, (**c**) Steel.

The reactant gas distribution across the catalyst layers is among the factors that could be used to examine a fuel cell performance due to its effect on the current density generated. A gas distribution that is uniform will prolong the membrane electrolyte assembly life-span and improve the overall performance of the fuel cell. In addition, bipolar material and the interaction with reactant supply to the catalyst layer for the electrochemical reaction will affect the cell performance. Here, different bipolar plate material at both electrodes catalyst layer with a constant operating voltage of 0.7V at a temperature of 323K and a pressure 1.5 bar is examined. Hydrogen is known to be the lightest element in the universe; for this reason; it diffuses in any environment readily. As can be seen in Figure 16 hydrogen concentration depleted at the middle of the channel where the catalyst layer is located, which was consumed for an electrochemical reaction from the inlet to the outlet fluid channel for the materials as a result of consumption of reactant due to reaction. On closer inspection, it is revealed that Al and Cu material followed a similar trend, in the sense that the consumption rate of hydrogen for Al material was slightly higher than Cu and SS material. The oxygen mole fraction for the different materials is shown in Figure 17. It can be seen that the oxygen mole fraction rate is high at the inlet channels for all the various materials and decreases gradually throughout the channels to the outlet channels due to the electrochemical reaction taking place at the cathode side of the fuel cell. It is noticed that the oxygen consumption rate for Al material is more than other materials. The more the consumption rate of reactant the more the current will be generated. 

**Figure 18:**
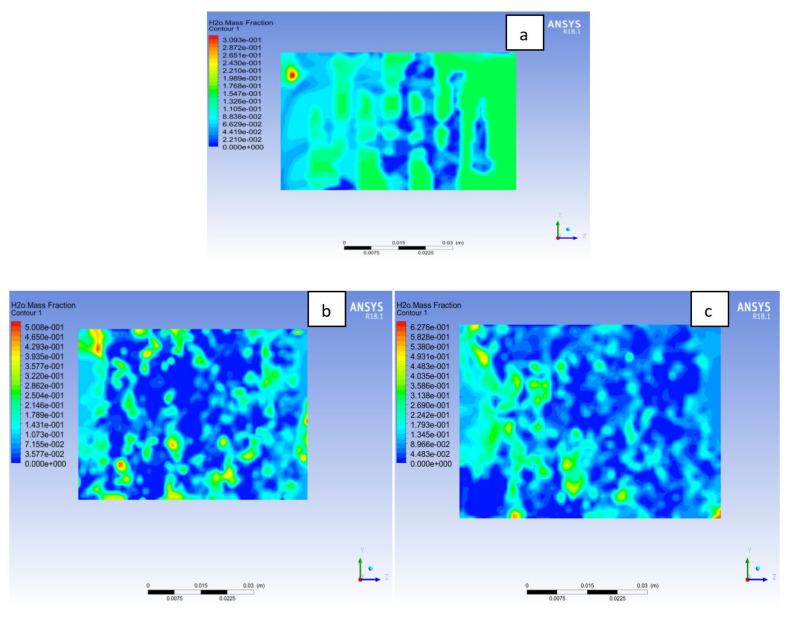
Water mass fraction at the membrane for (**a**) Aluminum, (**b**) Copper, (**c**) Steel bipolar plate materials at 338 K, 1.5 bar.

The membrane needs to be sufficiently hydrated so that the ionic transport from anode to cathode side will not be hindered. However, too much hydration of the membrane could lead to flooding of the MEA, which would affect the fuel cell performance. In Figure 18 the water on membrane mass fraction distribution is at cell potential 0.7V. It is noticed that the water content in the membrane is more in the Al material than Cu and SS materials. A particular amount of water is needed for membrane hydration, and it improves the ionic conductivity across the membrane, which will influence the fuel cell power output. It is also observed that Cu and SS material has more zones where the membrane is experiencing dryness than Al material as a result of activation losses or temperature of the cell. More water uniformity in the membrane can be found in the Al material than its counterpart, which will contribute to the overall cell performance. It is beneficial for the water distribution to be uniform in order to avoid the membrane from flooding or dry-out, which affect cell performance negatively [39,40,41]. 

### 4.4. Modeling Results Validation

**Figure 19:**
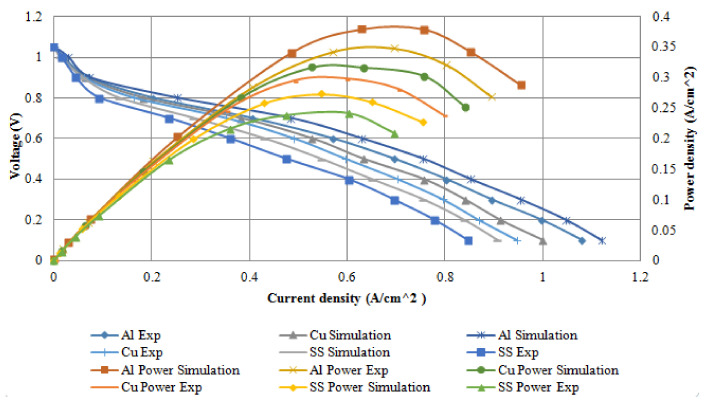
Comparison between numerical and experimental results of various bipolar plate materials.

The modeling results in this study were compared with the experimental results of Shimpalee et al. [40]. Figure 19 present the comparison between the modeling findings and experimental polarisation curve results of the fuel cell performance. In the experiments of Shimpalee et al. [40], a PEM fuel cell of bipolar plate materials of stainless steel 304, 430 L, and graphite material was compared against one another, with an active area of 25 cm^2^. Some of these materials were integrated into Ansys software in order to simulate and find out the best material cell performance. In their experiment, a serpentine flow field plate was used with an anode and cathode parallel flow. A single cell with a serpentine flow channel with the co-counter flow was used in the model to represent their experimental setup.

Figure 19 illustrates serpentine bipolar plate materials of the polarisation curves (left y-axis) and power density (right y-axis) for both numerical and experimental results for the single fuel cell under the same operating conditions. It is observed that the materials followed a similar behaviour and the effect of each material at low current density can be negligible but at a higher current density, the effect of the material on cell performance is significant. Additionally, it is noticed that the simulation results performed better than the experimental results. The Al material performed better than Cu and SS throughout the operating condition which reflected both in the numerical and experimental results, which can be attributed to low mass transport losses. Yu et al. [42] investigated an experiment on adsorption of Al, Cu, Mg, and Ti surfaces. Their results show that hydrogen adsorption on Al surfaces is more stable than other metals. A similar experiment was conducted by Meng and Alfred [43]; they concluded that hydrogen atoms bond weakly to Cu surfaces. Since SS is an alloy metal; this contributes to the reason why it has the lowest fuel cell performances. In addition, SS surface interactions with hydrogen atoms will result in high instability. The peak powers percentage difference for various materials between simulation and experimental results are illustrated in Table 4.

## 5. Conclusions

A low cost bipolar plate materials with a high fuel cell performance are important for the establishment of PEM fuel cells into the competitive market world. A computational fluid dynamics (CFD) investigation was conducted to examine the effect of different bipolar plates materials of aluminum, copper and stainless steel on a single cell of proton exchange membrane (PEM) fuel cells performances. The solutions from the PEM fuel cell model of various variables and polarisation curves were derived. 

It was observed that the Al bipolar plate material temperature distribution in the fuel cell was the best and it has the lowest pressure drop than Cu and SS material. It is worth nothing, that in terms of hydrogen, oxygen, and water vapor distributions the Al bipolar plate shows a better uniformity; which will increase the ionic conductivity in the membrane. Similarly, the experimental results agreed with the simulation results showing that Al serpentine bipolar plate has the best overall PEM fuel cell performance. The findings is related to literature experimental results on adsorption of metal surfaces, which shows that hydrogen molecules is more stable on Al surface than Cu and SS surfaces. Hence, Al sepentine bipolar plate material can be considered to be used as the best bipolar plate materail, especially for portable applications due to it light weight and reasonable cheap price of material.

## Figures and Tables

**Table 1:** Geometry properties.

Parameters	Value	Unit
Current collector width (anode side)	45	mm
Current collector width (cathode side)	45	mm
Gas flow field channel width	45	mm
Gas flow field channel depth	2	mm
Cell electrode length	65	mm
Gas diffusion layer thickness (anode region)	0.39	mm
Gas diffusion layer thickness (cathode region)	0.39	mm
Catalyst layer thickness (anode side)	0.08	mm
Catalyst layer thickness (cathode side)	0.08	mm
Active area	25	cm^2^
Membrane thickness	0.6	mm
Gas diffusion layer porosity (anode side)	0.5	−
Gas diffusion layer porosity (cathode side)	0.5	−
Catalyst layer porosity (anode region)	0.5	−
Catalyst layer porosity (cathode region)	0.5	−

**Table 2:** Parameters for simulation.

Parameters	Value	Unit
Operating temperature	298/323/338	K
Operating pressure	1.5/2/2.5	Bar
Mole fractions for hydrogen and water vapor (anode region)	0.6/0.4	−
Mole fractions for oxygen and water vapor (cathode region)	0.2/0.15	−
Relative humidity at anode side	100	%
Relative humidity at cathode side	100	%
Open circuit voltage	0.7	V

**Table 4:** Peak power data is for simulation and experimental results for the three bipolar plate materials.

Materials	Peak Power (Simulation)	Peak Power (Experimental)	% Deviation b/w Simulation and Experimental Results
Aluminium	0.36	0.33	8.33
Copper	0.3	0.28	6.67
Steel	0.25	0.23	8.00
